# Prediction of gestational diabetes mellitus by different obesity indices

**DOI:** 10.1186/s12884-022-04615-0

**Published:** 2022-04-06

**Authors:** Zhimin Song, Yan Cheng, Tingting Li, Yongfang Fan, Qingying Zhang, Haidong Cheng

**Affiliations:** 1 Department of Gynecology, Women’s Hospital, Zhejiang University School of Medicine, Hangzhou, Zhejiang 310006 People’s Republic of China; 2 Obstetrics and Gynecology Hospital, Fudan University, 128 Shenyang Road, Shanghai, 200090 People’s Republic of China

**Keywords:** Gestational diabetes mellitus, Obesity indices, Abdominal circumference, Abdominal circumference height ratio, Receiver operator characteristic, First trimester pregnancy

## Abstract

**Background:**

The incidence rates of obesity and gestational diabetes mellitus (GDM) are increasing in parallel. This study aimed to evaluate the relationship between different obesity indices, including prepregnancy body mass index (preBMI), the first-trimester abdominal circumference (AC), and first-trimester abdominal circumference/height ratio (ACHtR), and GDM, and the efficacy of these three indices in predicting GDM was assessed.

**Methods:**

A total of 15,472 pregnant women gave birth to a singleton at the Obstetrics and Gynecology Hospital of Fudan University, Shanghai, China. Prepregnancy weight was self-reported by study participants, body height and AC were measured by nurses at the first prenatal visit during weeks 11 to 13^+6^ of pregnancy. GDM was diagnosed through a 75-g oral glucose tolerance test at 24–28 gestational weeks. Using receiver operator characteristic (ROC) curve analysis, we evaluated the association between obesity indices and GDM.

**Results:**

A total of 1912 women (12.4%) were diagnosed with GDM. Logistic regression analysis showed that AC, ACHtR, and preBMI (*P* < 0.001) were all independent risk factors for the development of GDM. In the normal BMI population, the higher the AC or ACHtR was, the more likely the pregnant woman was to develop GDM. The area under the ROC curve (AUC) was 0.63 (95% CI: 0.62–0.64) for the AC, 0.64 (95% CI: 0.62–0.65) for the ACHtR and 0.63 (95% CI: 0.62–0.64) for the preBMI. An AC ≥ 80.3 cm (sensitivity: 61.6%; specificity: 57.9%), an ACHtR of ≥ 0.49 (sensitivity: 67.3%; specificity: 54.0%), and a preBMI ≥ 22.7 (sensitivity: 48.4%; specificity: 71.8%) were determined to be the best cut-off levels for identifying subjects with GDM.

**Conclusions:**

An increase in ACHtR may be an independent risk factor for GDM in the first trimester of pregnancy. Even in the normal BMI population, the higher the AC and ACHtR are, the more likely a pregnant woman is to develop GDM. AC, ACHtR in the first trimester and preBMI might be anthropometric indices for predicting GDM, but a single obesity index had limited predictive value for GDM.

## Background

Gestational diabetes mellitus (GDM) is any degree of glucose intolerance with an onset or its first recognition during pregnancy, and hyperglycaemia that is initially detected at any time during pregnancy should be classified either as diabetes mellitus in pregnancy (DIP) or GDM [1, 2]. It is a carbohydrate-intolerant state and one of the fastest growing pregnancy complications. All pregnant women should be screened for GDM with a laboratory-based screening test(s) using blood glucose levels. Screening for GDM is generally performed at 24–28 weeks of gestation [3]. Early pregnancy screening for undiagnosed type 2 diabetes, preferably at the initiation of prenatal care, is suggested in overweight and obese women with additional diabetes risk factors, including those with a prior history of GDM. The use of historic factors (family or personal history of diabetes, previous adverse pregnancy outcome, glycosuria, and obesity) to identify GDM will fail to identify approximately half of women with GDM [3]. The utility of first trimester fasting glycaemia is limited due to its low accuracy for GDM prediction [4]. The cause of the disease is complex [5]. Obesity is an independent high-risk factor for GDM [6, 7]. The incidence of GDM in China is as high as 17.5% [6]. Obese pregnant women are more likely to have GDM, preeclampsia, gestational hypertension, depression, instrument deliveries and caesarean sections, as well as surgical site infections. GDM is also related to the risk of premature delivery, large for gestational age infants, foetal defects and perinatal death. From 1993 to 2009, the prevalence of overweight increased from 10.7% to 14.4% among women (*P* < 0.001), and the prevalence of general obesity increased from 5.0% to 10.1% (*P* < 0.001) [8]. In the US, the incidence of prepregnancy obesity increased by approximately 70.0% between 1993 and 2003, showing a significant growth trend [9]. The definition of obesity in pregnancy has not been standardized. On the one hand, due to physiological changes during pregnancy, a woman's weight increases significantly in a relatively short period of time, and most of the weight gain (the foetus, amniotic fluid, blood, etc.) in pregnancy is immediately lost after delivery; on the other hand, across different regions and ethnic groups, there are differences in the diagnostic standards of obesity. Therefore, body mass index (BMI) before pregnancy is often used to define whether pregnant women are obese. Based on the differences in weight between the Chinese population and Western population, according to the recommendation of the Chinese adult BMI classification published by the China Obesity Working Group in 2001, mothers are categorized into four groups: low weight (BMI < 18.5), normal weight (18.5 ≤ BMI < 24.0), overweight (24.0 ≤ BMI < 28.0) and obese (BMI ≥ 28.0) [10]. However, the evaluation of obesity during pregnancy using BMI has certain limitations. BMI does not distinguish whether body weight comes from fat, muscle or other components and dose not accurately reflect the fat content and fat distribution across the human body, which is of great significance for clinical evaluation. Therefore, in addition to BMI, other indicators of obesity, such as waist circumference, neck circumference, waist/height ratio, waist/hip ratio, and body fat ratio, have been used to evaluate and predict the occurrence of GDM. Different obesity indicators are differentially effective in measuring and predicting gestational diabetes [11, 12]. According to the available research evidence, the incidence rates of obesity and GDM are rising in parallel. It is meaningful to explore the relationship between obesity and GDM. The international guidelines on gestational diabetes or gestational obesity are mostly based on characteristics of Western populations. Considering race and ethnicity, regional environment, differences in lifestyles and diets, and trends in economic development [13, 14], along with an increasing incidence of both obesity and GDM, there is a need to make a  better prevention and management of gestational obesity and GDM through large data research based on the Chinese population.

## Methods

### Study population

The Human Research Ethics Committee of the Obstetrics and Gynecology Hospital of Fudan University approved this retrospective study. The data were collected from the Obstetrics and Gynecology Hospital of Fudan University from January 1, 2017, to June 30, 2019. A total of 15,472 singleton pregnant women with complete prenatal care services and delivery in this hospital had data available for analysis. Patients with pregestational diabetes mellitus, severe medical complications and tumours were excluded.

### Clinical characteristics

All of the data were extracted from medical charts and the hospital information system. Clinical characteristics (including age, gravidity, parity and weight) were registered by self-report and/or measured at the first prenatal visit in the first trimester (11 ~ 13^+6^ weeks gestation), and delivery data were recorded in the electronic medical record system.

### Anthropometric measurements

Prepregnancy weight was self-reported by study participants, body height and AC (to the nearest 0.1 cm) were measured by nurses at the first prenatal visit during weeks 11 to 13^+6^ of pregnancy. PreBMI was calculated as prepregnancy weight (kg) divided by the square of body height (m). Women were categorized into four groups based on preBMI in accordance with the recommendation of the Chinese adult BMI classification published by the China Obesity Working Group in 2001. Before the measurement of abdominal circumference (AC), the pregnant women emptied their bladders, lay on their backs, and straightened their legs; then, a soft ruler was used to measure the distance around the abdomen at the level of the navel. The minimum circumference was recorded to the nearest 0.1 cm. Two trained nurses who had completed a training program obtained the anthropometric measurements, and the abdominal circumference/height ratio (ACHtR) was calculated by the abdominal circumference (m) divided by the height(m).

The pregnant women were classified in one of three groups according to the AC quartiles (low-AC, Q1 [AC < 74.0 cm, *n* = 3270]; normal-AC, Q2/Q3 [74.0 cm ≤ AC ≤ 86.0 cm, *n* = 8687]; and high-AC, Q4 [AC > 86.0 cm, *n* = 3515]) or ACHtR quartiles (low ACHtR, Q1 [ACHtR < 0.46, *n* = 3985]; normal ACHtR, Q2/Q3 [0.46 ≤ ACHtR ≤ 0.53, *n* = 7776]; and high ACHtR, Q4 [ACHtR > 0.53, *n* = 3711]). The low AC or ACHtR group was defined as the participants in Q1, the normal AC or ACHtR group was defined as those in Q2 and Q3, and the high AC or ACHtR group was defined as those in Q4. Because the normal range of AC or ACHtR has not yet been established, cut-off values for AC and ACHtR were not clearly defined.

### GDM diagnosis

The 75-g OGTT has been defined as the gold standard for GDM diagnosis, and the diagnostic criteria were based on the International Association of Diabetes and Pregnancy Study Groups (IADPSG). Women were considered to have GDM when any one or more of the following values equalled or exceeded these thresholds: FPG, 5.1 mmol/L; 1-h plasma glucose,10.0 mmol/L; 2-h plasma glucose; 8.5 mmol/L [15, 16]. The subjects were divided into the GDM group (*n* = 1912) or the control group (*n* = 13,560).

### Statistical analysis

All analyses were performed using SPSS for Windows version 24.0 (SPSS Inc., Chicago, IL, USA) with statistical significance set at 2-sided *p* < 0.05. Continuous variables are presented as the mean (SD), and skewed variables are described as the median (interquartile range). We used t tests for independent samples that were normally distributed continuous variables, and chi squared (χ^2^) tests were used for categorical variables. Receiver operator characteristic (ROC) curve analysis was performed to evaluate the ability of the ACHtR, AC and preBMI to predict GDM, and the Youden index, equivalent to the maximum sum of the sensitivity and specificity for all possible values of the cut-off point, which was defined as *J* = *sensitivity* + *specificity -1* [17]. Logistic regression analysis (using the backwards method) was conducted to explore the independent risk factors associated with GDM and the odds ratios (ORs) with 95% confidence intervals (CIs) associated with different obesity indices.

## Results

Our study included 15,472 women, consisting of 1912 women with GDM and 13,560 women without GDM. The clinical characteristics of the participants are shown in Table 1. The GDM group was older, included a greater proportion of multiparas and multigravid women, and delivered in earlier gestational weeks (*P* < 0.001). The mean AC (84.2 vs. 80.2 cm, ACHtR (0.52 vs. 0.50) and preBMI (22.9 vs. 21.5 kg/m2) were higher in the GDM group than in the control group (*P* < 0.001). However, neonatal birth weight and proportion experiencing postpartum haemorrhage were similar in the 2 groups (*P* > 0.05).

**Table 1:** Characteristics of the GDM group and control group^a^

	GDM *n* = 1912	Control *n* = 13,560	*P*
Age (years)	32.0 ± 4.1	30.5 ± 3.8	< 0.001
PreBMI (kg/m^2^)	22.9 ± 3.6	21.5 ± 3.0	< 0.001
< 18.5	17.6 ± 0.7	17.6 ± 0.7	0.59
≥ 18.5 to < 24.0	21.0 ± 1.5	21.4 ± 1.5	< 0.001
≥ 24.0 to < 28.0	25.7 ± 1.1	25.5 ± 1.1	< 0.001
≥ 28.0	30.5 ± 2.3	30.0 ± 1.9	0.008
AC (cm)	84.2 ± 9.4	80.2 ± 8.8	< 0.001
Q1 (< 74.0)	70.2 ± 2.7	70.0 ± 2.6	0.45
Q2/Q3(74.0–86.0)	80.3 ± 3.6	79.4 ± 3.6	< 0.001
Q4 (> 86.0)	94.3 ± 6.4	93.6 ± 6.2	0.009
ACHtR (cm/cm)	0.52 ± 0.06	0.50 ± 0.05	< 0.001
Q1 (< 0.46)	0.44 ± 0.02	0.44 ± 0.02	0.15
Q2/Q3(0.46–0.53)	0.50 ± 0.02	0.49 ± 0.02	< 0.001
Q4 (> 0.53)	0.58 ± 0.04	0.58 ± 0.04	0.09
Parity (%)			< 0.001
Primipara	1349(70.6)	10,277(75.8)	
Multipara	563(29.4)	3283(24.2)	
Gravidity (%)			< 0.001
Primigravid	846 (44.2)	7224 (53.3)	
Multigravid	1066 (55.8)	6336 (46.7)	
Gestational weeks of delivery	38.6 ± 1.5	38.9 ± 1.5	< 0.001
Neonatal birth weight (kg)	3.32 ± 0.49	3.34 ± 0.45	0.09
Postpartum hemorrhage (%)	53(2.8)	312(2.3)	0.12

^a^*PreBMI* Prepregnancy body mass index, *AC* Abdominal circumference, *ACHtR* Abdominal circumference/height ratio

Values are mean (SD) or medians (interquartile ranges). BMI were divided into four groups: low weight(BMI < 18.5), normal weight (18.5 ≤ BMI < 24.0), overweight (24.0 ≤ BMI < 28.0) and obese (BMI ≥ 28.0); AC were divided into three groups according to the quartile(Q1, low-AC group(< 74.0 cm);Q2/Q3, normal-AC group(74.0–86.0 cm); Q4, high-AC group(> 86.0 cm); ACHtR were divided into three groups according to the quartile (Q1, Low- ACHtR group(< 0.46);Q2/Q3, Normal-ACHtR group(0.46–0.53); Q4, High-ACHtR group(> 0.53))

Logistic regression analysis (Table 2) showed that in the normal BMI population (*n* = 10,382), the risk of GDM increased with increasing AC. The risk of GDM in the high AC group was 1.5 times higher than that in the normal AC group (OR = 1.50; 95% CI, 1.26–1.80), and the risk of GDM in the low AC group was 0.5 times than that in the normal AC group (OR = 0.52; 95% CI, 0.43–0.65). The ACHtR results were similar. The incidence risk of GDM in the high ACHtR group was 1.5 times higher than that in the normal group (OR = 1.54; 95% CI, 1.31–1.83), while the incidence risk of GDM in the low ACHtR group was 0.5 times than that in the normal ACHtR group (OR = 0.48; 95% CI, 0.40–0.58).

**Table 2:** Relative risk of GDM in those with normal BMI (*n* = 10,382) across the different AC and ACHtR groups^a^

	GDM/Total (%)	OR(95%CI)	*P*
AC (cm)			
Q1 (< 74.0)	106/1748 (6.1)	0.52(0.43–0.65)	< 0.001
Q2/Q3 (74.0–86.0)	825/7526 (11.0)	1	< 0.001
Q4 (> 86.0)	173/1108 (15.6)	1.50(1.26–1.80)	< 0.001
ACHtR			
Q1 (< 0.46)	133/2315 (5.7)	0.48(0.40–0.58)	< 0.001
Q2/Q3 (0.46–0.53)	770/6840 (11.3)	1	< 0.001
Q4 (> 0.53)	201/1227 (16.4)	1.54(1.31–1.83)	< 0.001

^a^*AC* Abdominal circumference, *ACHtR* Abdominal circumference/height ratio

*OR* Odds ratio, AC were divided into three groups according to the quartile (Q1, low-AC group (< 74.0 cm);Q2/Q3, normal-AC group (74.0–86.0 cm); Q4, high-AC group (> 86.0 cm); ACHtR were divided into three groups according to the quartile (Q1, Low- ACHtR group (< 0.46);Q2/Q3, Normal-ACHtR group (0.46–0.53); Q4, High-ACHtR group (> 0.53))

**Fig. 1:**
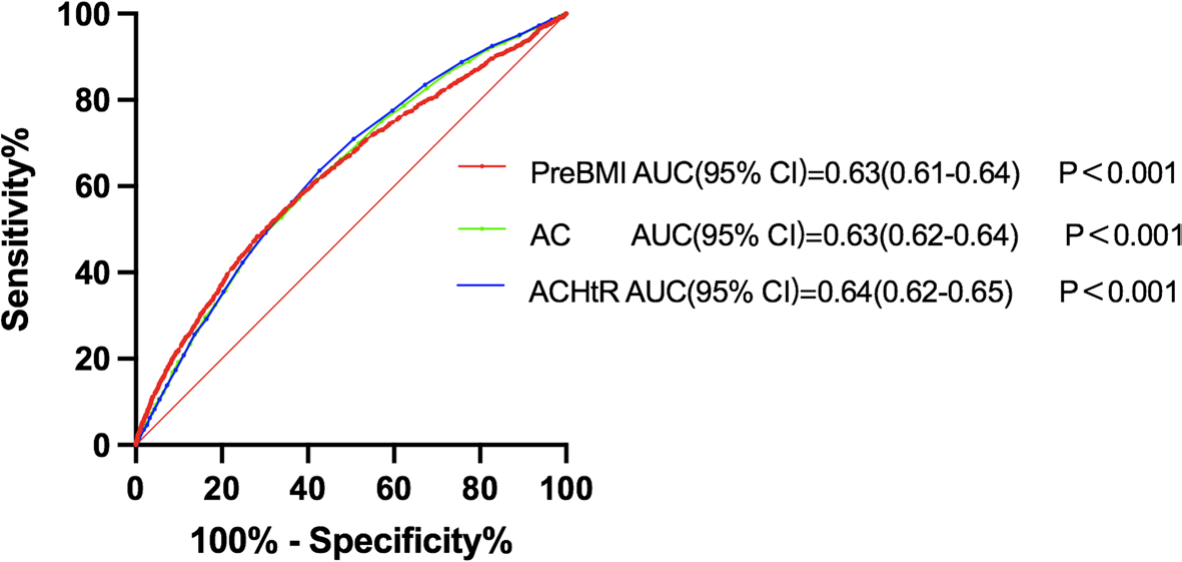
ROC curves for preBMI, AC and ACHtR in predicting GDM. PreBMI: prepregnancy body mass index; AC: abdominal circumference; ACHtR: abdominal circumference/height ratio

The ROC curve determined the ability of the ACHtR, AC and preBMI measures to identify GDM. The AUCs were 0.64 (95% CI, 0.63–0.65) with the ACHtR, 0.63 (95% CI, 0.62–0.64) with the AC, and 0.63 (95% CI, 0.62–0.64) with the preBMI measures (Fig. 1).

The results showed that the ACHtR and AC measures were similar to the preBMI measue in predicting GDM.

The optimal cut-off point was the point on the ROC curve closest to the (0, 1) point. An AC of 80.3 cm yielded the highest combination of sensitivity (61.6%) and specificity (57.9%), an ACHtR of 0.49 had a sensitivity of 67.3% and specificity 54.0%, and a preBMI of 22.7 yielded the highest combination of sensitivity (48.4%) and specificity (71.8%) (Table 3).

**Table3:** PreBMI, AC and ACHtR as predictors for gestational diabetes mellitus

	cut-off point	sensitivity	specificity	Youden index
PreBMI	22.7	48.4%	71.8%	0.202
AC(cm)	80.3	61.6%	57.9%	0.195
ACHtR	0.49	67.3%	54.0%	0.213

*PreBMI* Prepregnancy body mass index, *AC* Abdominal circumference, *ACHtR* Abdominal circumference/height ratio

Youden index formula is defined as *J* = *sensitivity* + *specificity -1*

Logistic regression analysis showed that, the ACHtR, preBMI, and AC were independent risk factors for GDM development. Model 1 was adjusted for age, gravidity, parity. The risk of GDM in the high AC group was 1.7 times higher than that in the normal AC group (OR = 1.70; 95% CI, 1.53–1.90), and the risk of GDM in the low AC group was 0.6 times than that in the normal AC group (OR = 0.58; 95% CI, 0.50–0.68); the incidence risk of GDM in the obese group was 3.1 times higher than that in the normal weight group (OR = 3.06; 95% CI, 2.53–3.70), while the incidence risk of GDM in the overweight group was 1.9 times higher than that in the normal weight group (OR = 1.87; 95% CI, 1.66–2.10). The incidence risk of GDM in the high ACHtR group was 1.7 times higher than that in the normal group (OR = 1.68; 95% CI, 1.51–1.90), while the incidence risk of GDM in the low ACHtR group was 0.6 times than that in the normal ACHtR group (OR = 0.58; 95% CI, 0.50–0.68); Model 2 was adjusted for age, gravidity, parity and height. The risk of GDM in the high AC group was 1.7 times higher than that in the normal AC group (OR = 1.72; 95% CI, 1.54–1.91), and the risk of GDM in the low AC group was 0.6 times than that in the normal AC group (OR = 0.57; 95% CI, 0.49–0.66); the incidence risk of GDM in the obese group was 1.9 times higher than that in the normal weight group (OR = 1.86; 95% CI, 1.45–2.38), while the incidence risk of GDM in the overweight group was 1.4 times higher than that in the normal weight group (OR = 1.42; 95% CI, 1.22–1.65) (Table 4).

**Table 4:** Relationship of preBMI, AC, or ACHtR with gestational diabetes mellitus

Variables	GDM/Total (%)	Model 1		Model 2	
		OR(95%CI)	*P*	OR(95%CI)	*P*
PreBMI^a^		1.13(1.12–1.15)	< 0.001	1.13 (1.12–1.15)	< 0.001
< 18.5	164/2021 (8.1)	0.82(0.69–1.98)	0.027	0.99(0.83–1.20)	0.987
≥ 18.5 to < 24.0	1104/10382 (10.6)	1		1	
≥ 24.0 to < 28.0	472/2456 (19.2)	1.87(1.66–2.10)	< 0.001	1.42 (1.22–1.65)	< 0.001
≥ 28.0	172/613 (28.1)	3.06 (2.53–3.70)	< 0.001	1.86 (1.45–2.38)	< 0.001
AC ^b^ (cm)		1.04 (1.03–1.05)	< 0.001	1.04 (1.04–1.05)	< 0.001
Q1 (< 74.0)	211/3270 (6.5)	0.58(0.50–0.68)	< 0.001	0.57(0.49–0.66)	< 0.001
Q2/Q3 (74.0–86.0)	1015/8687 (11.7)	1		1	< 0.001
Q4 (> 86.0)	686/3515 (19.5)	1.70(1.53–1.90)	< 0.001	1.72(1.54–1.91)	< 0.001
ACHtR		1.07 (1.06–1.07)*	< 0.001	N/A	
Q1 (< 0.46)	253/3985 (6.3)	0.56(0.48–0.64)	< 0.001	N/A	
Q2/Q3 (0.46–0.53)	929/7776 (11.9)	1		N/A	
Q4 (> 0.53)	730/3711 (19.7)	1.68(1.51–1.90)	< 0.001	N/A	

Logistic regression analysis of ACHtR, AC, or preBMI with confounders of gestational diabetes mellitus; p*reBMI* prepregnancy Body mass index, *AC*: Abdominal circumference, *ACHtR* Abdominal circumference/height ratio, *CI* Confidence interval, *OR* Odds ratio, *N/A* Not applicable

Model 1 adjusted for age, gravidity and parity

^a^Model 2 as Model 1 plus AC

^b^Model 2 as Model 1 plus height

^*^Based on percentile of abdominal circumference/height ratio

## Discussion

With the development of the economy and the improvement of national living standards, the incidence rates of GDM and obesity are increasing year by year. BMI before pregnancy, weight gain at different stages of pregnancy, and gestational weight gain are clearly associated with adverse pregnancy outcomes [18]. Previous studies have shown that an increase in maternal obesity (BMI and other obesity-related indicators) increases the risk of GDM. Increases in BMI before pregnancy are linearly related to the incidence rate of GDM, with the probability of occurrence of GDM being increased by 0.9% with a 1.0 kg/m^2^ increase in BMI, and obese older women (BMI > 23.0 and age > 35) were more likely to be affected by GDM [7, 19, 20]. An average annual weight gain between 20 and 24 years old of more than 1.5% with a BMI within a particular range is also an important risk factor for GDM [21]. An increase in body weight ≥ 2.5 kg/year during the 5 years before being pregnant may increase the risk of GDM by 2.5 times [22]. With a previous pregnancy with a normal BMI and a second pregnancy with a BMI of classified as obese, there is a 3.0-fold increase in risk of gestational diabetes, and weight loss during the two pregnancies could reduce the risk of gestational diabetes mellitus [23]. In addition to BMI, other obesity evaluation indices have been used to evaluate and predict the occurrence of GDM. An increase in neck circumference in early pregnancy may be one of the independent risk factors for GDM. When NC ≥ 33.8 cm in early pregnancy, the incidence of GDM was shown to be significantly increased [24, 25]. Abdominal subcutaneous fat thickness (ASFT) can be measured by ultrasound at 10 ^+ 6^ to 13 ^+ 6^ weeks of gestation, and an ASFT is ≥ 2.4 cm predicts the risk and prognosis of GDM in middle and late pregnancy [25, 26]. Visceral adipose tissue thickness (VAT) and total adipose tissue thickness (TAT) assessed by ultrasonography in early pregnancy can independently predict the risk of abnormal blood glucose in late pregnancy: with increases in VAT and TAT, the incidence of diabetes increases [27].

In our present study, we demonstrated that ACHtR in the first trimester could be used as a novel indicator to predict GDM. Obesity, especially abdominal obesity (AO), has been considered a risk factor for diabetic complications. Abdominal obesity may be defined as excess deposits of fat in the abdominal region. It has been positively related to noncommunicable diseases, such as cardiovascular diseases, diabetes, hypertension, cancer, kidney diseases and nonalcoholic fatty liver disease. The latest guidelines for South Asians define AO as large waist circumference (WC), i.e., ≥ 90.0 cm in men and ≥ 80.0 cm in women, independent of BMI [28]. According to a recent study, the waist/height ratio (WHtR) is better than WC in predicting AO [29]. However, pregnant women generally do not have waist circumference measured during pregnancy, although abdominal circumference is measured to accurately estimate the size of the foetus. Therefore, we used abdominal circumference and the abdominal circumference/height ratio to evaluate abdominal obesity in pregnant women. Our research suggests that pregnant women with a higher AC or ACHtR and normal BMI also have a higher risk of GDM, which shows that even in women with a normal BMI, the prevalence of GDM is higher in individuals with abdominal obesity. The diagnostic accuracy of AC and ACHtR in the first trimester for predicting GDM was similar to that of preBMI. An AC of 80.3 cm and ACHtR of 0.49 might be the optimal cut-off points for predicting GDM. Although some studies have shown that AC is one of the risk factors for GDM, and the combination of AC and other variables was used to build an early model to predict GDM [30, 31]; however, the sample sizes in these studies were not particularly large, or there was a need for invasive tests. Whether ACHtR is correlated with GDM remains unclear, and there is a lack of relevant studies. In our study, the AUC was 0.63 for AC and 0.64 for ACHtR in predicting GDM, and these findings were better than the traditional predictor, preBMI, with an AUC of 0.63 in predicting GDM. Because abdominal circumference and height are objective data measured by doctors, they are more objective measures than when pregnant women recall their weight before pregnancy, and these measures can avoid the possibility of memory errors or subjective errors. In fact, some women cannot accurately state their prepregnancy weight.

The optimal cut-off point of an AC of 80.3 cm yielded the highest combination of sensitivity and specificity, and the cut-off points were an ACHtR value of 0.49 and a preBMI of 22.7; these data indicated that AC and the ACHtR might be good anthropometric indices to screen for GDM. However, different studies may report different sensitivities and specificities at different cut-off points, possibly due to different study populations, gestational weeks and ethnicities. A recent study supported that the optimal cut-off level of maternal WC in the first trimester of pregnancy was > 84.5 cm, with a sensitivity of 78.0% and a specificity of 54.0%. Another study found that WC can predict GDM in the range of 86.0–88.0 cm at 20–24 weeks of gestation [32]. Larger sample studies and multicentre studies are needed to determine the optimal cut-off values for AC, preBMI, and the ACHtR to predict GDM.

Previous papers have utilized patients' biometric data for the early prediction of GDM by creating logistic regression models and performing ROC analysis. A study published in 2021 employed a 7-variable logistic regression (LR) model and achieved effective discriminatory power (AUC = 0.77) [33]. Another prospective cohort study that included 1385 pregnant women showed that the risk of GDM in women with BMI 24–28 kg/m2 in the first trimester was 1.9 times that in normal weight women (95% CI, 1.20–2.91, *p* < 0.05). The risk of GDM in women with BMI > 28 kg/m2 was 4.5 times that in women with normal weight (95% CI, 2.07–9.82, *p* < 0.05). Although their results seem to be slightly better than ours, our study used only objective measures of the body, which were very easy to obtain, and did not require any invasive examination. In addition, we included a relatively large sample size.

There were also some limitations in our study. On the one hand, this study was a single-centre study in Shanghai, China, which may have affected the results and restricted the generalization of the study conclusions. Further studies needs to be conducted to confirm our findings. On the other hand, all subjects were asked to retrospectively report their weight prior to pregnancy, which was used to calculate preBMI. As the study began after delivery, It was difficult to obtain accurate measurements of prepregnancy weights.

## Conclusion

In this study, we found that a higher ACHtR may be an independent risk factor for GDM in the first trimester of pregnancy. Even in the normal BMI population, the higher the AC and ACHtR were, the more likely a pregnant woman was to be diagnosed with GDM. AC, and ACHtR in the first trimester and preBMI might be anthropometric indices for predicting GDM, but a single obesity index has limited predictive value for GDM.

## Data Availability

The datasets generated and/or analysed during the current study are not publicly available due patient privacy but are available from the corresponding author on reasonable request.

## Abbreviations

GDM: Gestational diabetes mellitus
DIP: Diabetes mellitus in pregnancy
preBMI: Prepregnancy Body Mass Index
AC: Abdominal circumference
ACHtR: Abdominal circumference/height ratio
ROC: Receiver operator characteristic
AUC: Area under the curve
AO: Abdominal obesity
WC: Waist circumference
NC: Neck circumference
VAT: Visceral adipose tissue thickness
TAT: Total adipose tissue thickness
ASFT: Abdominal subcutaneous fat thickness
